# A new category of autoinflammatory disease associated with NOD2 gene mutations

**DOI:** 10.1186/ar3462

**Published:** 2011-09-14

**Authors:** Qingping Yao, Lan Zhou, Philip Cusumano, Nilanjana Bose, Melissa Piliang, Bijal Jayakar, Le-Chu Su, Bo Shen

**Affiliations:** 1 Department of Rheumatic and Immunologic Diseases, Cleveland Clinic, 9500 Euclid Avenue, Cleveland, OH 44195, USA; 2 Neurology, Cleveland Clinic, 9500 Euclid Avenue, Cleveland, OH 44195, USA; 3 General Medicine, Cleveland Clinic, 9500 Euclid Avenue, Cleveland, OH 44195, USA; 4 Dermatology and Anatomic Pathology, Cleveland Clinic, 9500 Euclid Avenue, Cleveland, OH 44195, USA; 5 Gastroenterology/Hepatology, Cleveland Clinic, 9500 Euclid Avenue, Cleveland, OH 44195, USA

## Abstract

**Introduction:**

Autoinflammatory diseases are characterized by seemingly unprovoked episodes of inflammation, without high titers of autoantibodies or antigen-specific T cells, and derive from genetic variants of the innate immune system. This study characterized a cohort of patients with similar phenotypes and nucleotide oligomerization domain 2 (NOD2) gene mutations.

**Methods:**

Diagnostically challenging patients with the following clinical and genetic characteristics were prospectively studied between January 2009 and April 2011: periodic fever, dermatitis, polyarthritis, serositis, negative serum autoantibodies and additional positive NOD2 IVS8^+158 ^gene mutation. Genetic testing for gene mutations of NOD2, tumor necrosis factor receptor-associated periodic fever syndrome (TRAPS) and familial Mediterranean fever (FMF) was performed.

**Results:**

All seven patients with the disease were Caucasians, with four being male. The mean age at disease onset was 40.7 years and disease duration was 3.2 years. These patients characteristically presented with periodic fever, dermatitis and inflammatory polyarthritis. There were gastrointestinal symptoms in three patients, granulomas of the skin and gut in two, and recurrent chest pain in two, with one having pleuritis and pericarditis. Three patients had sicca-like symptoms. Five patients had increased acute phase reactants. All seven patients had negative tests for autoantibodies but carried the NOD2 gene mutation IVS8^+158 ^with four having concurrent R702W mutation.

**Conclusions:**

Our cohort may represent a new disease category of autoinflammatory disease with characteristic clinical phenotypes and genotypes. It may somewhat resemble pediatric Blau's syndrome.

## Introduction

Autoinflammatory diseases (AIDs) were initially defined as seemingly unprovoked episodes of inflammation, without high titer autoantibodies or antigen specific T cells [1]. It is recently proposed that the AIDs are clinical disorders marked by abnormally increased inflammation, mediated predominantly by the cells and molecules of the innate immune system, with a significant host predisposition [2]. AIDs represent a wide disease spectrum, ranging from Mendalian disorders such as Blau's syndrome (BS) to a more complex (polygenic) mode of inheritance such as Crohn's disease [2]. In clinical practice, particularly in tertiary referral centers, physicians may encounter various autoinflammatory phenotypes which could cause highly costly and unnecessary repetitive workups. We hypothesized these phenotypes could be associated with unidentified gene mutations. Herein, we report an adult case series with similar clinical phenotypes to support a novel AID entity associated with positive nucleotide oligomerization domain (NOD2) gene mutations.

## Materials and methods

### Patients

Seven diagnostically complex patients with symptoms of multiple system involvement were referred to our Rheumatology Clinic at the Cleveland Clinic between January 2009 and April 2011. These adult patients had undergone extensive evaluations from multidisciplinary departments. This prospective study was approved by the Institutional Review Board.

### Laboratory evaluation

In addition to routine blood tests, all patients also had special tests for systemic autoimmune diseases including, but not limited to, classic connective tissue diseases and systemic vasculitis. The serum autoantibodies tested included antinuclear antibodies, anti-extractable nuclear antigen (Sm, RNP, SSA, SSB, Scl70, centromere, Jo-1 and chromatin) antibodies, rheumatoid factor, anti-citrullinated peptide antibodies, anti-dsDNA antibodies, complements 3 and 4, lupus anticoagulant, anti-cardiolipin antibodies, anti-beta2glyprotein I antibodies and anti-neutrophil cytoplasmic antibodies.

Since these patients were clinically suspected of autoinflammatory diseases, such as BS, blood specimens of the patients were sent and genetically tested by the Center for Genetic Testing in Saint Francis, Oklahoma, USA for NOD2 gene mutations after informed consent was obtained. Examination of the NOD2 gene for mutations was performed by DNA polymerase chain reactions and DNA sequencing of all 12 coding exons. In addition, genetic testing for tumor necrosis factor receptor associated periodic fever syndrome (TRAPS) and familial Mediterranean fever (FMF) was also conducted in some cases as clinically appropriate (GeneDx, Gaithersburg, Maryland, USA).

### Inclusion and exclusion criteria

The inclusion clinical criteria were patients having 1) periodic fever, which was defined as episodic fever of unknown origin two or more times during the disease process; 2) dermatitis; 3) polyarthritis; 4) serositis; and 5) absence of any autoantibodies. The disease entity was considered to be present if three or more criteria were met plus additional positive NOD2 IVS8^+158 ^gene mutation. We also excluded systemic autoimmune diseases and other AIDs.

### Outcome measurement

Demographics, and clinical and genetic features of the patients were characterized. Descriptive statistics were used.

## Results

A total of seven patients were included in the study. All were Caucasians, with four being men (57.1%). The mean age at disease onset was 40.7 years (range 21 to 60 years) and disease duration was 3.2 years (range 1 to 8 years). No family history of any AIDs was reported.

### Constitutional symptoms

Constitutional symptoms included flu-like symptoms, significant weight loss and fatigue. Five patients had periodic fevers with each episode lasting a few days to several weeks and varying afebrile intervals. Table 1 summarizes the demographic, clinical and genotypic features of the seven patients.

**Table 1:** Demographic, clinical and subclinical features of seven patients

Patient	1	2	3	4	5	6	7
Gender	Male	Female	Female	Male	Female	Male	Male
Age at diagnosis (years)	41	45	53	28	62	37	40
Disease duration(years)	1	1	8	7	2	2	1
Ethnicity	White	White	White	White	White	White	White
Weight loss	Yes	Yes	No	Yes	Yes	Yes	Yes
Fever	Yes	No	No	Yes	Yes	No	Yes
Skin disease	Yes	Yes	Yes	Yes	Yes	Yes	No
Polyarthritis	Yes	Yes	Yes	No	Yes	Yes	No
Serositis	No	No	No	No	No	Chest pain	Pleuritis/pericarditis
Ocular	Normal	Blurry, dry	Blurry, dry	Normal	Normal	Pain and redness, dry	No
Gastrointestinal	Normal	Pain, diarrhea	Normal	Pain	Normal	Pain and diarrhea, lipasemia	Elevated LFT^a^
Familial	No	No	No	No	No	No	No
ESR^b ^(mm/hr)	61	5	60	92	10	2	74
CRP^c ^(mg/dl)	7.5	NA^d^	17.9	7.2	12.3	Normal	38.7
NOD2^e^	IVS8+^158^ C > T, R702W	IVS8+^158^ C > T, R702W	IVS8+^158^ C > T	IVS8+^158^ C > T	IVS8+^158^ C > T, R702W	IVS8+^158^ C > T R702W	IVS8+^158^ C > T
TRAPS^f^	NA	Negative	NA	Negative	Negative	Negative	Negative
MEFV^g^	NA	NA	NA	NA	Negative	NA	Negative

^a^Liver enzymes; ^b^erytherocyte sedimentation rate; ^c^c-reactive protein; ^d^not available; ^e^nucleotide oligomerization domain; ^f^tumor necrosis factor receptor associated periodic fever syndrome; ^g^Mediterranean fever

### Cutaneous presentation

**Figure 1:**
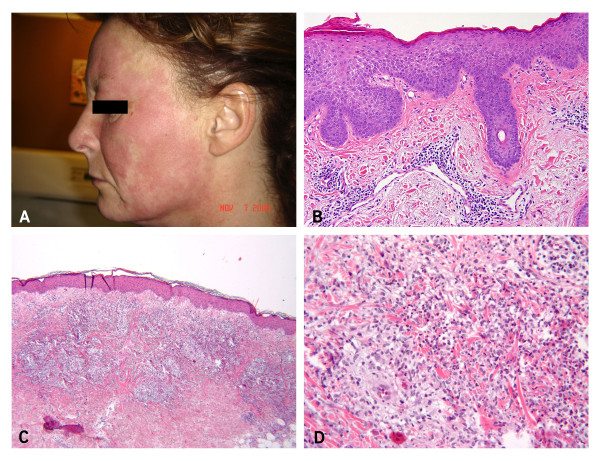
**Skin manifestation**. Erythematous edematous plaques in Patient 2 (**A**), subacute spongiotic dermatitis in Patient 2 (**B**), 40×, granulomatous dermatitis in Patient 3 (**C **and **D**), Hematoxylin and eosin, 20× and 40×.

Six of the seven patients had skin disease presented with pruritic or nonpruritic erythematous edematous plaques, patches, macules, papules and linear scratch-like rash in the face (Figure 1A), chest, abdomen and limbs. Among these patients, two had spongiotic dermatitis, with superficial perivascular lymphohistocytic (Patient 1), lymphoplasmacytic infiltrate (Patient 2, Figure 1B), and Patient 5 had mixed lymphocytic and neutrophilic parivascular dermatitis. Patient 3 had perivascular and mixed inflammatory infiltrate which consisted of numerous activated histocytes forming ill-defined granulomas and rare associated multinucleated cells, consistent with palisaded neutrophilic and granulomatous dermatitis (Figure 1C, D). There were capillaritis without evidence of vasculitis (Patient 4) and oral ulcers (Patient 6).

### Inflammatory arthritis

Multiple joint pain, tenderness and swelling were common and the arthritis was non-erosive. Polyarthralgia can affect nearly any joints in the limbs, particularly the hip, knee and ankle. Hip symptoms were prominent in three cases. Patient 3 underwent bilateral total hip replacement in her 40s presumably due to osteoarthritis.

### Gastrointestinal (GI) manifestation

Three patients presented with intermittent GI symptoms, such as abdominal pain and/or diarrhea, but without radiographic, endoscopic or histologic evidence of Crohn's disease. Patient 4 presented with recurrent fever, abdominal pain without diarrhea and abnormal liver enzymes and was found to have mild colitis in the cecum and sigmoid colon, with gastric and colonic nonnecrotizing granulomas. A computerized tomography (CT) scan and positron emission tomography scan showed numerous enlarged mesenteric lymph nodes, histologically consistent with granulomatous lymphadenitis. Patient 6 developed two episodes of abdominal pain and nausea with transient lipasemia suspicious of acute idiopathic pancreatitis.

### Cardiopulmonary manifestations

Patient 6 also complained of recurrent chest pain with negative cardiopulmonary and GI workups. Patient 7 had recurrent chest pain associated with pleuritis on chest radiographs and pericarditis on echocardiography.

### Ophthalmic and neurological manifestations

Three of the patients complained of blurry vision and dry eyes but ophthalmologic examination revealed central scotoma of both eyes, right eye episcleritis and a stable choroidal nevus in the left eye in Patient 3. There was no uveitis found in these three patients. These sicca-like symptoms prompted a workup for Sjögren's syndrome but antinuclear antibodies, anti-SSA/anti-SSB antibodies and minor salivary gland biopsies all were negative. Neurological symptoms included headaches in three cases but with an unremarkable neurological examination. One patient (Patient 2) presented with paresthesias of the extremities and later was found to have small fiber sensory neuropathy.

### Laboratory and genetic testing results

Patient 1 had mild anemia, leukocytosis and eosinophilia. Five patients had elevated acute phase reactants. The special tests for systemic autoimmune diseases were negative as stated in the methods. Urinalysis was negative in all patients and chest radiographs and CT were negative in all but Patient 7. Two patients (Patients 1 and 4) underwent hematological evaluation but with normal results. All seven patients were confirmed to carry the NOD2 gene mutations, including IVS8^+158 ^in all patients and R702W in four (Table 1). Patients 2, 4, 5, 6 and 7 also had negative gene testing for TRAPS and/or FMF.

### Therapy and outcomes

Our patients were empirically treated with nonsteroidal antiinflammatory drugs (NSAIDs), prednisone, hydroxychloroquine and TNF-α blockers. NSAIDs appeared ineffective for arthritic manifestation, which responded to a low dose prednisone (< 20 mg daily). Two of the patients were taking sulfasalazine two to three grams daily for arthritis for 3 to 12 months with minimal improvement. One patient continued to have bilateral hip pain despite taking a small dose prednisone, sulfasalazine and methotrexate. The patients with skin disease responded to prednisone but not to hydroxychloroquine. One patient with recurrent pleuritis/pericarditis did not respond to colchicine or NSAIDs but responded to high dose prednisone. One patient was treated with TNF-α blockers (infliximab and adalimumab) with a partial response for inflammatory arthritis and fevers. Among the three patients with GI symptoms, one (Patient 4) continued to have intermittent abdominal pain and the other two had no abdominal pain and diarrhea recently.

## Discussion

AIDs are characterized by seemingly unprovoked episodes of inflammation, without high titer autoantibodies or antigen specific T cells, and derive from genetic variants of the innate immune system, including Mendalian and genetically complex disorders [1]. The disease entity we describe in the present paper consists of periodic fevers, skin disease, inflammatory arthritis, serositis, sicca-like symptoms and elevated acute phase reactants but the hallmarks of autoimmunity are lacking, therefore it is congruent with an AID. This constellation of clinical phenotypes together with the NOD2 gene mutation may constitute a variant of the AIDs. To our knowledge, this disease entity does not fit any known AIDs. Given its strong association with the gene mutation, we presently categorize and designate this disease as a new AID. We believe that this condition may be underdiagnosed due to a lack of awareness of this clinical entity. This disease entity could represent a polygenic disease because it did not appear rare. This disorder seems similar to pediatric BS in phenotypes and genotypes to some extent, thus we will focus the following discussion on a Blau's-like syndrome as well as Crohn's disease.

BS (MIM186580) is an autosomal dominant AID, which was originally characterized by a triad of granulomatous dermatitis, arthritis and uveitis [3]. Currently, BS and early onset sarcoid arthritis are accepted as the same disease [4]. There have been over 154 cases of pediatric BS reported, involving 41 families [5]. Other manifestations include fevers, abnormal liver function tests, large vessel arteritis, cranial neuropathy, pneumonitis, lymphadenitis, sialadenitis, erythema nodosum [5,6] and sinus of valsalva aneurysm [7]. To the best of our knowledge, there have been no reports of adult onset cases of BS. Our case series study demonstrates that the clinical manifestations of this new adult disease entity are partially within the reported clinical spectrum of pediatric BS; however, the disease entity also differs from pediatric BS in that the former may not present with the classic triad of the pediatric form and has some distinct clinical features as discussed below.

First, we found that periodic fevers, dermatitis and polyarthralgias/inflammatory polyarthritis are common in our case series but without deformity, Camptodactyly (flexion contracture of the fingers and toes), that was originally reported in the members of a single family by Blau [3]. Second, while pediatric BS can present with a variety of skin manifestations [5], our adult patients showed predominantly non-granulomatous dermatitis with occasional granulomas. Third, uveitis has been reported in 61% (46/76) of the pediatric BS patients [8]; however, none of our patients had uveitis.

Among the seven patients, there were three cases of GI symptoms which prompted an exhaustive search for Crohn's disease. One patient carried a history of questionable Crohn's disease, but symptoms of periodic fevers and skin disease with minimal evidence of active Crohn's disease would argue against the likelihood of Crohn's disease in this patient. Although granulomas are known to occur in both Crohn's disease and BS [9], non-granulomatous dermatitis as in our series is extremely rare in Crohn's disease [10]. Nevertheless, these patients did not have cogent evidence of Crohn's disease. Therefore, an intermediate subset of the new AID with Crohn's disease-like presentation ought to be entertained in these patients and Crohn's disease should be listed as a differential diagnosis.

The NOD2 gene mutations (chromosome 16q12-21) have been principally linked to both Crohn's disease and BS [11-14]. To date, there are 105 NOD2 gene mutation sequence variants reported [15]. NOD2 IVS8^+158 ^gene mutation was initially reported to confer higher risk for Crohn's disease in Ashkenazi Jews [16]. Subsequently, two available studies failed to replicate the association between the gene mutation and Crohn's disease in the Ashkenazi Jews [17] and Jewish Israeli population [18]. None of our patients were Jewish. It is worth noting that this gene mutation was not studied and reported in pediatric BS until it was recently reported to associate with the disease in a single case report where Borzutzky A, *et al*. [19] described a nine-month-old Caucasian boy who developed fever, migratory rash consistent with cutaneous tuberculoid granulomas and inflammatory arthritis. In conjunction with granulomatous panuveitis, the patient was diagnosed with early onset sarcoidosis/Blau's syndrome, and the NOD2 gene mutation IVS8^+158 ^was found. In our series, the IVS8^+158 ^NOD2 gene mutation was detected in all patients. Interestingly, four patients with positive IVS8^+158 ^also carried the R702W gene mutation. These findings suggest that the combined IVS8^+158 ^and R702W gene mutations may confer a higher risk for the currently described new AID. Of note, there were cases of similar clinical presentations with negative NOD2 gene mutations in our study. For example, one adult case of Sjögren's syndrome and one case of adult onset sarcoidosis without pulmonary involvement had negative NOD2 gene mutations. To the best of our knowledge, there have been no reports on the involvement of IVS8^+158 ^in other diseases apart from Crohn's disease in Ashkenazi Jews and BS to date. We presently propose that the gene mutation IVS8^+158 ^may be entertained as a diagnostic marker for the new entity. However, the clinical relevance of such gene mutations to these diseases remains to be determined given the limited studies. We anticipate more investigations, such as analysis of haplotype frequency, DNA sequencing of the entire gene and the genotype-phenotype correlation in the new AID using genome wide association study.

## Conclusions

Our adult case study suggests that the currently described disease entity constitutes a new category of AIDs, which phenotypically resembles the pediatric BS cases and can mimic Crohn's disease. Genotypically, the IVS8^+158 ^NOD2 mutation appears associated with the disease, particularly in conjunction with R702W. This new disease entity may be genetically complex rather than Mendalian.

## Abbreviations

AIDs: autoinflammatory diseases; BS: Blau's syndrome; CT: computerized tomography; FMF: familial Mediterranean fever; GI: gastrointestinal; NOD2: nucleotide oligomerization domain; NSAIDs: nonsteroidal anti-inflammatory drugs; TRAPS: tumor necrosis factor receptor associated periodic fever syndrome.

## Competing interests

The authors declare that they have no competing interests.

## Authors' contributions

YQ contributed to the conception and design of the study. YQ, ZL, CP, BN, PM, JB and SL participated in data collection. YQ, ZL, PM and SB participated in data analysis and interpretation, and drafting and reviewing of the manuscript. All authors have read and approved the manuscript for publication. Written consent to publish was obtained from the patients.
